# Short- and long-term performance of risk calculation tools for mortality in patients with acute coronary syndrome

**DOI:** 10.3389/fcvm.2024.1388686

**Published:** 2024-05-28

**Authors:** Takatoku Aizawa, Tomoaki Nagao, Yusuke Oda, Suguru Nakano, Kazuki Ito, Yusuke Shirai, Natsuko Hosoya, Kohei Sawasaki, Junji Arai, Shinya Fujita, Masahiro Muto, Teiji Oda, Yuichiro Maekawa

**Affiliations:** ^1^ Department of Cardiology, Hamamatsu Medical Center, Hamamatsu, Japan; ^2^ Division of Cardiology, Department of Internal Medicine Ⅲ, Hamamatsu University of Medicine, Hamamatsu, Japan

**Keywords:** time-dependent receiver operating characteristic analysis, mortality, risk calculation tool, ACTION score, CRUSADE score, GRACE score, acute coronary syndrome, predictive performance

## Abstract

**Background:**

The mortality rate of acute coronary syndrome (ACS) remains high. Therefore, patients with ACS should undergo early risk stratification, for which various risk calculation tools are available. However, it remains uncertain whether the predictive performance varies over time between risk calculation tools for different target periods. This study aimed to compare the predictive performance of risk calculation tools in estimating short- and long-term mortality risks in patients with ACS, while considering different observation periods using time-dependent receiver operating characteristic (ROC) analysis.

**Methods:**

This study included 404 consecutive patients with ACS who underwent coronary angiography at our hospital from March 2017 to January 2021. The ACTION and GRACE scores for short-term risk stratification purposes and CRUSADE scores for long-term risk stratification purposes were calculated for all participants. The participants were followed up for 36 months to assess mortality. Using time-dependent ROC analysis, we evaluated the area under the curve (AUC) of the ACTION, CRUSADE, and GRACE scores at 1, 6, 12, 24, and 36 months.

**Results:**

Sixty-six patients died during the observation periods. The AUCs at 1, 6, 12, 24, and 36 months of the ACTION score were 0.942, 0.925, 0.889, 0.856, and 0.832; those of the CRUSADE score were 0.881, 0.883, 0.862, 0.876, and 0.862; and those of the GRACE score 0.949, 0.928, 0.888, 0.875, and 0.860, respectively.

**Conclusions:**

The ACTION and GRACE scores were excellent risk stratification tools for mortality in the short term. The prognostic performance of each risk score was almost similar in the long term, but the CRUSADE score might be a superior risk stratification tool in the longer term than 3 years.

## 1 Introduction

Compared with the early days, the clinical outcomes of percutaneous coronary intervention (PCI) for ischemic heart disease have seen significant improvement (1). Advancements in PCI techniques and devices have reached a mature stage (2, 3). However, the mortality rate of acute coronary syndrome (ACS) remains high, despite the era of third-generation drug-eluting stents (4, 5). Therefore, patients with ACS should undergo early risk stratification (6), for which various risk calculation tools are available (7–16). Early risk stratification helps with determining treatment strategies for ACS. The widely used risk calculation tools are the TIMI and GRACE risk scores (15, 17). While several studies have compared the performance of risk calculation tools (18–27), no consensus has been reached on which risk calculation tool is superior. One potential reason could be the difference in the observation periods of various studies. Furthermore, the risk scores for estimating short- and long-term prognosis have been proposed. Whether the predictive performance of risk calculation tools varies over the long term remains unclear.

Receiver operating characteristic (ROC) curve analysis is commonly used to evaluate the predictive performance of continuous variables for a single endpoint event. However, conventional ROC analysis cannot integrate the effects of time into the evaluation. Previous studies have relied on conventional ROC analyses to compare the predictive performance of risk calculation tools. However, the observation duration could significantly affect the study results and mortality rates. If the analysis is performed after considering the time course, dropout cases must be considered. Time-dependent ROC analysis enables the analysis of the predictive performance of independent variables for the occurrence of endpoints while considering dropout cases over time (28). Therefore, evaluating the predictive performance of independent variables for event outcomes by incorporating the time course using a time-dependent ROC analysis is meaningful.

This study aimed to compare the predictive performance of risk calculation tools to estimate short- and long-term mortality risk in patients with ACS while considering different observation periods using time-dependent ROC analysis.

## 2 Methods

### 2.1 Study population

This single-center retrospective study included consecutive patients with ACS who underwent coronary angiography at Hamamatsu Medical Center from March 2017 to January 2021. The exclusion criteria were cases with missing data for each risk calculation tool or the absence of severe stenosis or thrombus in the epicardial coronary artery on coronary angiography.

This study adhered to the principles of the Declaration of Helsinki and was approved by the Medical Ethics Committee of Hamamatsu Medical Center (2023-3-037). As this was a retrospective study, obtaining informed consent from each patient was not required. Instead, in accordance with our institution's routine ethical regulations, we posted a notice on the study design and contact information in a public location within our institution. In this public notification, we ensured that patients had the opportunity to opt out of participating in this study.

### 2.2 Clinical endpoints

The endpoint of this study was all-cause mortality. We evaluated the occurrence of endpoints at 1, 6, 12, 24, and 36 months after coronary angiography.

### 2.3 Risk calculation tools

All participants were scored using three widely used risk calculation tools: ACTION, CRUSADE, and GRACE. The ACTION risk score (range: 0–103) includes age, systolic blood pressure, creatinine clearance, cardiac arrest, shock, heart failure, heart rate, ST-segment elevation myocardial infarction (STEMI), and troponin ratio (11). The CRUSADE risk score (range: 0–142) includes age, serum creatinine level, systolic blood pressure, heart failure, heart rate, weight, prior heart failure, hematocrit level, troponin ratio, prior stroke, diabetes mellitus, sex, and history of peripheral artery disease (16). The GRACE risk score (range: 1–372) includes Killip class, systolic blood pressure, heart rate, age, serum creatinine level, cardiac arrest, ST-segment deviation, and elevated cardiac enzyme level (15).

### 2.4 Definitions and data collection

Myocardial infarction (MI) is associated with cardiac troponin release and is based on the third universal definition of MI (29). STEMI is defined as MI with ST-segment elevation ≥1 mm in at least two contiguous leads or a new or undetermined duration of left branch bundle block on electrocardiogram. Non-STEMI (NSTEMI) is defined as MI without the aforementioned electrocardiographic changes. Unstable angina is defined as myocardial ischemia at rest or with minimal exertion in the absence of acute cardiomyocyte injury or necrosis. Shock is defined as 90 mmHg systolic blood pressure or less at admission. Heart failure is defined as Killip class II, III, or IV on admission. The troponin ratio is calculated as the baseline troponin value divided by the local laboratory-specific upper limit of normal. The data required for the risk calculation tool were defined with reference to each study (11, 15, 16). Upon admission, all participants were subjected to medical history taking, physical examination, blood sampling, chest radiography, and electrical cardiography. Patients with STEMI underwent emergent coronary angiography, and those with non-ST-segment elevation ACS underwent emergent or elective coronary angiography, depending on the situation at admission. The decision regarding the therapeutic strategy was left to the attending physician's discretion, depending on the severity of the case. We collected data on the endpoints using information from the electronic medical records of our hospital and requests for written medical information from referral medical institutions but not by telephone contact with the patients directly. All extracted data were anonymized.

### 2.5 Statistical analysis

The continuous variables were expressed as mean ± standard deviation or median values with the associated range. The categorical variables were expressed as percentages. A comparison of baseline characteristics between the death and non-death groups was performed using the *t*-test and *χ*^2^ test for continuous and categorical variables, respectively. The Kaplan–Meier curve represented the occurrence of events after coronary angiography. Time-dependent ROC analysis was performed to evaluate the predictive performance of risk scores. Individual disease outcomes were observed and updated in the time-dependent ROC curve analysis at each time point. This curve was created using the sensitivity (*t*) and 1-specificity (*t*) obtained from various cutoff values of the independent variables at time *t*. Using the measured values, a time-dependent ROC curve could be drawn at any time *t*. The predictive ability of independent variables can be accurately evaluated by constructing ROC curves at several time points. Therefore, time-dependent ROC curve analysis is an efficient statistical method for precisely evaluating the outcomes of independent variables (28). *P* < 0.05 was considered statistically significant. All statistical analyses were performed using EZR (Saitama Medical Center, Jichi Medical University, Saitama, Japan), a graphical user interface for R (R Foundation for Statistical Computing, Vienna, Austria) (30). Specifically, it is a modified version of the R commander designed to add statistical functions frequently used in biostatistics. We used the Kaplan–Meier “survival ROC” package, written in R, to assess time-dependent changes in prediction performance between different risk calculation tools (28). Furthermore, we performed additional analysis using the “timeROC” package in R to evaluate the 95% confidence intervals of the area under the time-dependent ROC curves and the statistical difference between the area under the time-dependent ROC curves of risk scores (31).

## 3 Results

**Figure 1:**
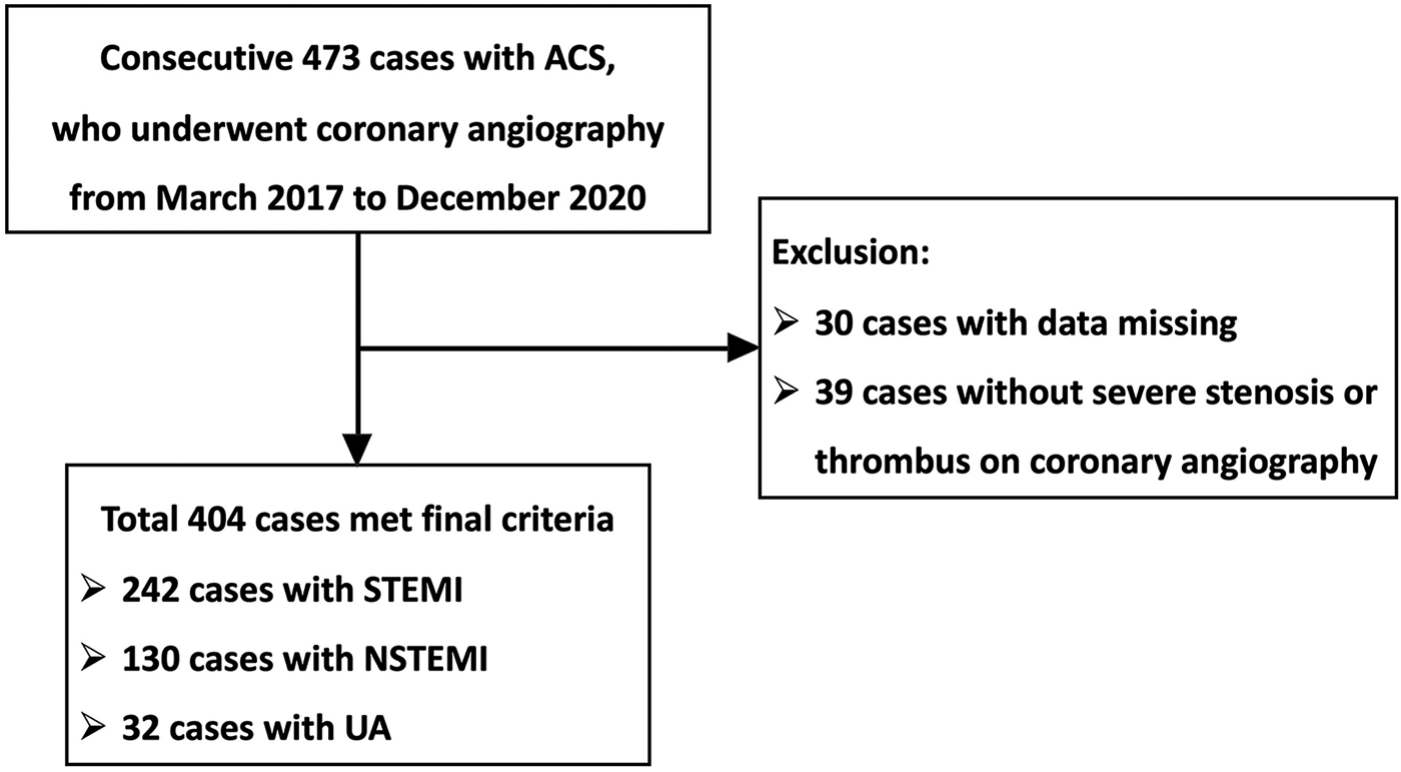
Flow diagram for the study population. ACS, acute coronary syndrome; STEMI, ST-segment elevation myocardial infarction; NSTEMI, non-ST-segment elevation myocardial infarction; UA, unstable angina.

Figure 1 illustrates the patient flow diagram detailing the progression through this study. The study population consisted of 473 consecutive patients with ACS who underwent coronary angiography at Hamamatsu Medical Center from March 2017 to January 2021. Thirty cases had missing data, and 39 did not have severe stenosis or thrombus on coronary angiography. Finally, we investigated the predictive performance of the risk calculation tools for 404 cases. In total, 242 patients (60.0%) had STEMI, 130 (32.1%) had NSTEMI, and 32 (7.9%) had unstable angina. We followed up with participants for 36 months.

### 3.1 Clinical characteristics

The baseline characteristics of the study population are listed in Tables 1, 2. Significant differences between most variables contributed to risk scores in the death and non-death groups. Among these variables, sex, heart rate, diagnosis of STEMI, and troponin ratio were not significantly different between the death and non-death groups.

**Table 1:** Clinical characteristics ①.

Variables	Death (*n* = 66)	Non-death (*n* = 338)	*P*-value
Age (year)	79.2 ± 10.1	69.5 ± 12.3	<0.001
Sex (male)	51 (77.3)	255 (75.4)	NS
Systolic blood pressure (mmHg)	112.6 ± 51.1	137.4 ± 33.0	<0.001
Heart rate (beats/min)	73.9 ± 32.8	76.2 ± 34.3	NS
Body weight (kg)	56.5 ± 13.0	63.1 ± 13.1	<0.001
Cardiac arrest	14 (21.2)	8 (2.4)	<0.001
Shock	29 (43.9)	26 (7.7)	<0.001
Heart failure	45 (68.2)	51 (15.1)	<0.001
Killip class			
I	20 (30.3)	288 (85.2)	<0.001
Ⅱ	11 (16.7)	23 (6.8)	
Ⅲ	6 (9.1)	1 (0.3)	
Ⅳ	29 (43.9)	26 (7.7)	
ST deviation of ECG	65 (98.5)	294 (87.0)	0.007
History			
Hypertension	49 (74.2)	210 (62.1)	NS
DM	36 (54.5)	132 (39.1)	0.02
Dyslipidemia	36 (54.5)	216 (63.9)	NS
Smoking	24 (36.3)	163 (48.2)	NS
Stroke	8 (12.1)	16 (4.7)	0.02
Heart failure	11 (16.7)	15 (4.4)	<0.001
PAD	7 (10.6)	12 (3.6)	0.013
Myocardial infarction	16 (24.2)	62 (18.3)	NS
Post PCI	15 (22.7)	61 (18.0)	0.049
Post CABG	0 (0)	3 (0.9)	NS
HD	5 (7.6)	6 (1.8)	0.008

NS, not significant; ECG, electrocardiogram; PAD, peripheral artery disease; PCI, percutaneous coronary intervention; CABG, coronary artery bypass grafting.

**Table 2:** Clinical characteristics ②.

Variables	Death (*n* = 66)	Non-death (*n* = 338)	*P*-value
Diagnosis			
STEMI	42 (63.6)	200 (59.2)	NS
NSTEMI	22 (33.3)	108 (32.0)	
UA	2 (3.0)	30 (8.9)	
Laboratory data			
Hematocrit (%)	37.0 ± 7.0	41.4 ± 5.4	<0.001
HbA1c (%)	6.4 ± 0.9	6.7 ± 2.2	NS
LDL-C (mg/dl)	107.4 ± 39.3	123.3 ± 38.1	0.004
HDL-C (mg/dl)	44.7 ± 11.3	49.9 ± 10.9	0.001
TG (mg/dl)	118.2 ± 99.3	125.6 ± 99.1	NS
Cr (mg/dl)	1.66 ± 1.69	1.11 ± 1.74	0.009
eGFR (ml/min/1.73 m^2^)	46.6 ± 24.7	66.7 ± 23.1	<0.001
Troponin I (pg/ml)	8,553.2 ± 22,955.3	4,352.7 ± 14,707.1	NS
Troponin ratio	434.9 ± 142.2	497.7 ± 860.0	NS
Number of vessel lesions of coronary angiography			
1 vessel lesion	30 (45.5)	208 (61.5)	0.015
2 vessel lesion	14 (21.2)	78 (23.1)	NS
3 vessel lesion	51 (15.1)	18 (27.3)	0.016
LMT lesion	9 (13.6)	25 (7.4)	NS
Therapeutic strategy			
PCI	59 (89.4)	314 (92.9)	NS
CABG	2 (3.0)	14 (4.1)	
Medical therapy	5 (7.6)	10 (3.0)	
Risk score			
ACTION risk score	57.7 ± 17.6	37.4 ± 11.8	<0.001
CRUSADE risk score	46.4 ± 13.7	26.1 ± 12.3	<0.001
GRACE risk score	208.2 ± 48.4	142.8 ± 36.8	<0.001

NS, not significant; STEMI, ST-segment elevation myocardial infarction; NSTEMI, non-ST-elevation myocardial infarction; HbA1c, hemoglobin A1c; LDL-C, low-density lipoprotein cholesterol; HDL-C, high-density lipoprotein cholesterol; TG, triglyceride; eGFR, estimated glomerular filtration rate; LMT, left main trunk; PCI, percutaneous coronary intervention; CABG, coronary artery bypass grafting.

### 3.2 Incidence rates of all-cause mortality

**Figure 2:**
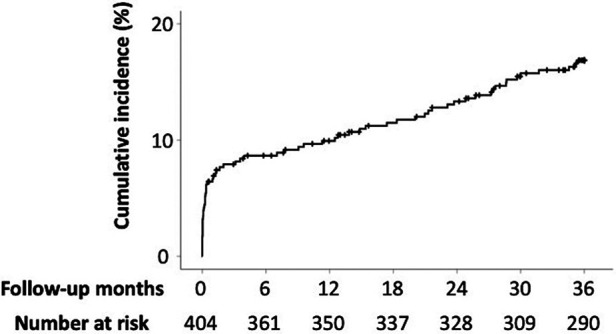
Incidence rates of all-cause mortality. The cumulative incidence rates for all-cause mortality at 1, 6, 12, 24, and 36 months are 6.7%, 8.7%, 9.9%, 13.3%, and 16.9%, respectively.

In total, 66 patients died during the 36-month follow-up period. The median follow-up period for the overall study was 1,080 days (interquartile range: 1,022–1,080). The cumulative incidence rates of all-cause mortality at 1, 6, 12, 24, and 36 months were 6.7%, 8.7%, 9.9%, 13.3%, and 16.9%, respectively (Figure 2).

### 3.3 Time-dependent ROC curves of the ACTION, CRUSADE, and GRACE risk scores for all-cause mortality

**Figure 3:**
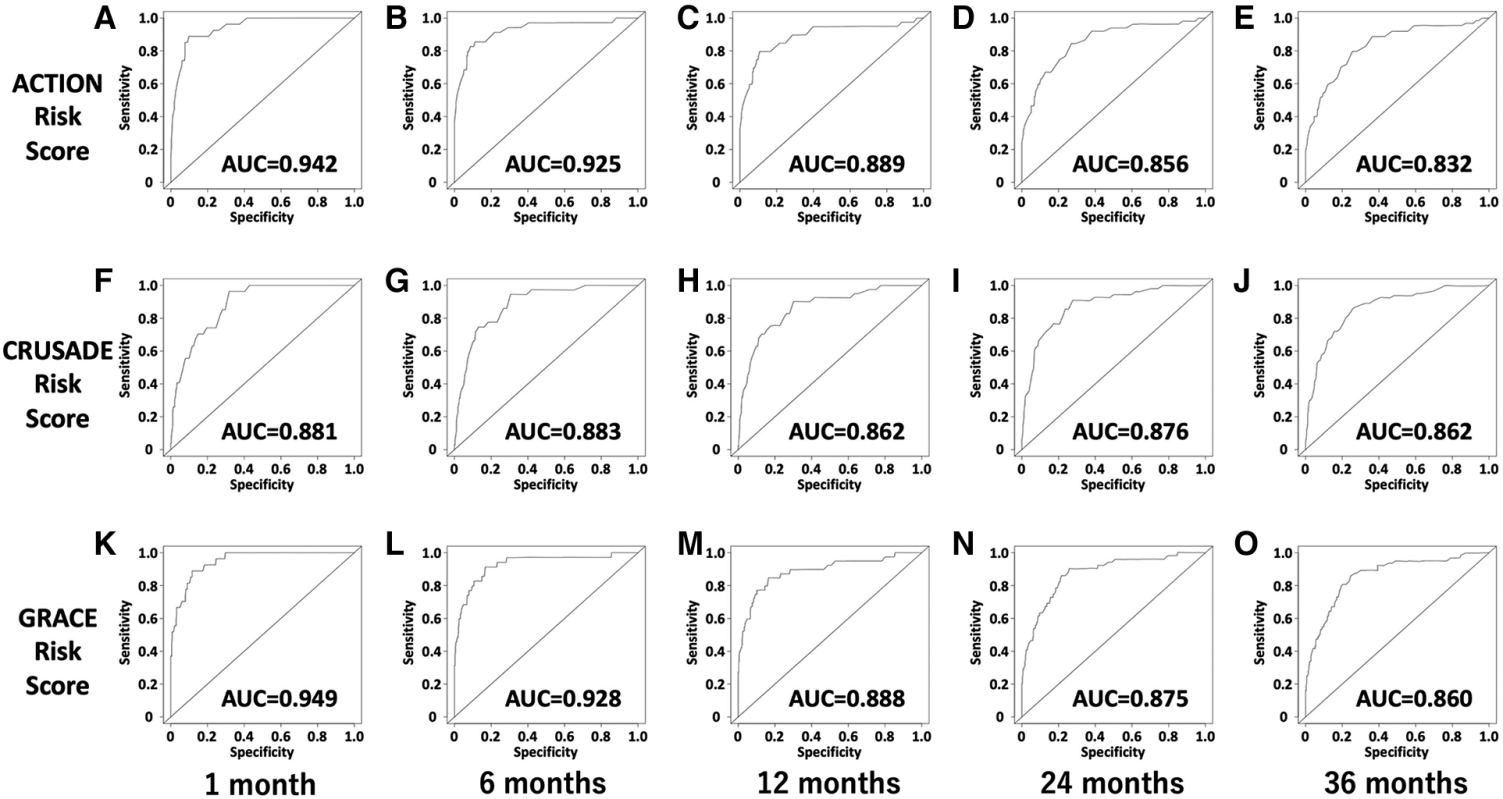
Time-dependent ROC curves of the ACTION, CRUSADE, and GRACE risk scores for all-cause mortality. (**A**) Time-dependent ROC curve of the ACTION risk score for all-cause mortality at 1 month. (**B**) Time-dependent ROC curve of the ACTION risk score for all-cause mortality at 6 months. (**C**) Time-dependent ROC curve of the ACTION risk score for all-cause mortality at 12 months. (**D**) Time-dependent ROC curve of the ACTION risk score for all-cause mortality at 24 months. (**E**) Time-dependent ROC curve of the ACTION risk score for all-cause mortality at 36 months. (**F**) Time-dependent ROC curve of the CRUSADE risk score for all-cause mortality at 1 month. (**G**) Time-dependent ROC curve of the CRUSADE risk score for all-cause mortality at 6 months. (**H**) Time-dependent ROC curve of the CRUSADE risk score for all-cause mortality at 12 months. (**I**) Time-dependent ROC curve of the CRUSADE risk score for all-cause mortality at 24 months. (**J**) Time-dependent ROC curve of the CRUSADE risk score for all-cause mortality at 36 months. (**K**) Time-dependent ROC curve of the GRACE risk score for all-cause mortality at 1 month. (**L**) Time-dependent ROC curve of the GRACE risk score for all-cause mortality at 6 months. (**M**) Time-dependent ROC curve of the GRACE risk score for all-cause mortality at 12 months. (***N***) Time-dependent ROC curve of the GRACE risk score for all-cause mortality at 24 months. (**O**) Time-dependent ROC curve of the GRACE risk score for all-cause mortality at 36 months. ACU, area under curve; ROC, receiver operating characteristics.

Figure 3 illustrates the ROC curves of each risk score for all-cause mortality at 1, 6, 12, 24, and 36 months after the start of follow-up, using time-dependent ROC analysis. Table 3 compares the areas under the curve (AUCs) of risk scores for all-cause mortality. The AUCs of the ACTION risk score at 1, 6, 12, 24, and 36 months were 0.942, 0.925, 0.889, 0.856, and 0.832, respectively; those of the CRUSADE risk score were 0.881, 0.883, 0.862, 0.876, and 0.862, respectively; and those of the GRACE risk score were 0.949, 0.928, 0.888, 0.875, and 0.860, respectively**.**

**Table 3:** Comparison between AUCs of risk scores for all-cause mortality.

			Period (month)	AUC (95% CI)		AUC (95% CI)	*P*-value
ACTION score	vs.	CRUSADE score	1	0.942 (0.905–0.978)	vs.	0.881 (0.833–0.932)	0.032
ACTION score	vs.	CRUSADE score	6	0.925 (0.870–0.980)	vs.	0.883 (0.832–0.934)	NS
ACTION score	vs.	CRUSADE score	12	0.889 (0.823–0.914]	vs.	0.862 (0.800–0.923)	NS
ACTION score	vs.	CRUSADE score	24	0.865 (0.798–0.914)	vs.	0.876 (0.824–0.924)	NS
ACTION score	vs.	CRUSADE score	36	0.832 (0.775–0.890)	vs.	0.862 (0.816–0.911)	NS
ACTION score	vs.	GRACE score	1	0.942 (0.905–0.978)	vs.	0.949 (0.917–0.980)	NS
ACTION score	vs.	GRACE score	6	0.925 (0.870–0.980)	vs.	0.928 (0.876–0.980)	NS
ACTION score	vs.	GRACE score	12	0.889 (0.823–0.914)	vs.	0.888 (0.825–0.954)	NS
ACTION score	vs.	GRACE score	24	0.865 (0.798–0.914)	vs.	0.875 (0.823–0.927)	NS
ACTION score	vs.	GRACE score	36	0.832 (0.775–0.890)	vs.	0.860 (0.811–0.912)	0.014
CRUSADE score	vs.	GRACE score	1	0.881 (0.833–0.932)	vs.	0.949 (0.917–0.980)	0.012
CRUSADE score	vs.	GRACE score	6	0.883 (0.832–0.934)	vs.	0.928 (0.876–0.980)	0.045
CRUSADE score	vs.	GRACE score	12	0.862 (0.800–0.923)	vs.	0.888 (0.825–0.954)	NS
CRUSADE score	vs.	GRACE score	24	0.876 (0.824–0.924)	vs.	0.875 (0.823–0.927)	NS
CRUSADE score	vs.	GRACE score	36	0.862 (0.816–0.911)	vs.	0.860 (0.811–0.912)	NS

AUC, area under curve; CI, confidence interval; vs, versus; NS, not significant.

### 3.4 Change in AUC of the ACTION, CRUSADE, and GRACE risk scores over time using time-dependent ROC analysis

**Figure 4:**
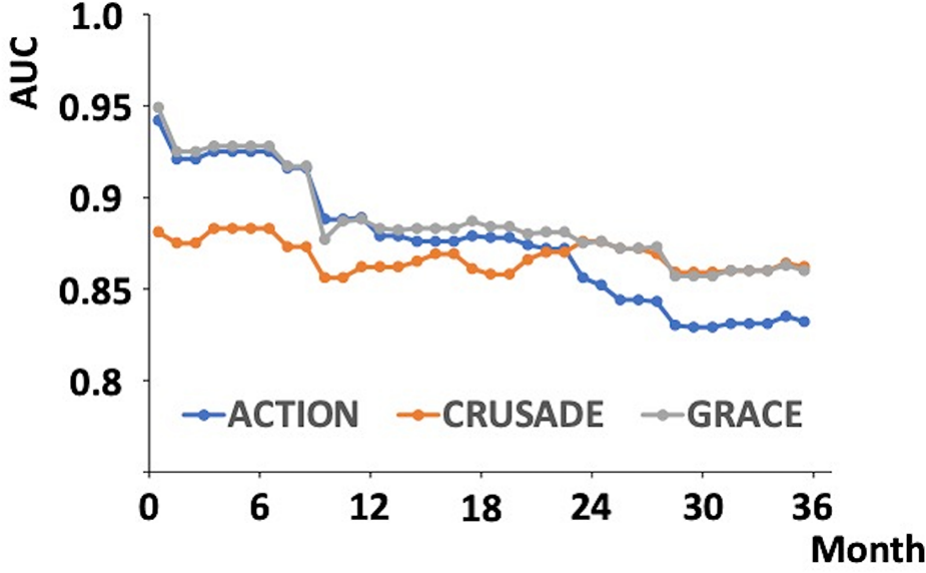
Change in AUC of the ACTION, CRUSADE, and GRACE risk scores over time using time-dependent ROC analysis. The AUCs of the ACTION, CRUSADE, and GRACE risks for all-cause mortality were plotted monthly from the start of follow-up to 36 months. ACU, area under curve; ROC, receiver operating characteristics.

Figure 4 depicts the AUC of each risk score for all-cause mortality and cardiac death plotted monthly using time-dependent ROC analysis. The AUCs of the ACTION and GRACE risk scores for all-cause mortality were higher than those of the CRUSADE score, especially in the short-term, but decreased over time. In contrast, the AUC of the CRUSADE score remained relatively constant during the observation period.

## 4 Discussion

### 4.1 Summary of results

This study evaluated the AUCs of the ACTION, CRUSADE, and GRACE risk scores for all-cause mortality using time-dependent ROC analysis for 36 months. The AUCs of each risk score for mortality in patients with ACS remained relatively high during the observation period. The AUCs of the ACTION and GRACE scores were significantly higher than that of the CRUSADE score in the short term but decreased over time. Conversely, the AUC of the CRUSADE score remained relatively constant.

### 4.2 Differences in AUC trends between risk calculation tools

The AUCs of the ACTION and GRACE risk scores were higher than those of the CRUSADE risk score, especially in the short term. However, the AUCs of the ACTION and GRACE risk scores decreased over time. Nevertheless, the AUC of the CRUSADE score remained relatively constant. Among the data used to calculate risk scores, only the CRUSADE risk score included sex, weight, diabetes mellitus, prior heart failure, prior stroke, and prior peripheral artery disease. The association between diabetes and arteriosclerotic disease is widely known; however, diabetes has also been reported to be associated with cancer, which greatly affects long-term prognosis (32–34). Furthermore, diabetes affects bone metabolism, and fractures in patients with diabetes worsen prognosis (35, 36). Other data for calculating the CRUSADE risk score also affect prognosis (37–42). Systolic pressure; cardiac enzyme levels; and the presence of heart failure, cardiac arrest, and cardiac shock, which are included in the non-CRUSADE risk scores or every risk score, are strongly associated with ACS severity. The mortality rate of ACS is highest during the acute phase (43). Therefore, the AUCs of the ACTION and GRACE risk scores, which have a larger proportion of data strongly associated with ACS severity, might be higher than that of the CRUSADE risk score in the short term and may decrease over time. In contrast, the AUCs of the CRUSADE risk score might have remained constant during the observation period because the CRUSADE risk score includes several data that affect prognosis, regardless of ACS severity.

### 4.3 The capability of other risk scores for long-term prognosis

This study used the CRUSADE risk score as the long-term risk calculation tool. However, other tools that target long-term prognosis, such as the ACEF, LASSO, and EPICOR scores, are also available (10, 12, 13). Because of the retrospective design of this study, accurate quantitative assessment of LVEF using an ultrasound cardiogram, the use of a diuretic agent, and the timing of the diuretic agent during hospitalization, which are included in these risk scores, were unclear. The left ventricular systolic function and the presence of heart failure might significantly impact long-term prognosis. Therefore, risk scores containing these parameters might demonstrate a more excellent long-term predictive performance than the CRUSADE risk score.

### 4.4 Clinical implications

Only a few studies have investigated the predictive performance of risk calculation tools for >1 year in patients with ACS. This is the first study to investigate 3-year time-dependent changes in predictive performance for mortality in patients with ACS between risk calculation tools to estimate short- and long-term risk, using time-dependent ROC analysis. The AUCs of each mortality risk score in patients with ACS remained relatively high during the observation period. This study revealed excellent risk stratification performance of the ACTION and GRACE risk scores, especially in the early phase. In contrast, the AUC of the CRUSADE risk score for all-cause death was reversed from the other risk scores during the 3-year observation period; however, there was no significant difference. The difference in the AUC between the CRUSADE and other risk scores might increase by extending the observation period to 5 or 10 years. The ACTION and GRACE risk scores are risk calculation tools proposed to estimate in-hospital mortality (11, 16). Conversely, the CRUSADE risk score is a risk calculation tool proposed to estimate long-term mortality (15). This study demonstrated that each risk score was suitable for risk stratification of the original target periods and was an excellent risk calculation tool. Therefore, the results of this study may support those of previous studies.

### 4.5 Study limitations

This study has a few limitations. This was a single-center, retrospective study. Furthermore, the sample size was small for testing and comparing several risk scores. Multicenter prospective studies are required to confirm the findings of this study. More than 40% of the patients had multivessel lesions or left main trunk lesions on coronary angiography, but only 3.7% underwent coronary artery bypass grafting as a therapeutic strategy. The results may differ in a facility with a better environment for emergency surgery. The study population was limited to patients who had undergone coronary angiography for ACS. High-risk cases in which ACS was suspected but coronary angiography was not performed because of advanced age, frailty, or bleeding complications may have been included. Patients who did not undergo coronary angiography may have a poorer prognosis. Cases without visible thrombus or critical stenosis, which is almost equivalent to myocardial infarction with non-obstructive coronary disease (MINOCA), were excluded. The inclusion of MINOCA cases might lead to different results.

## 5 Conclusions

The ACTION and GRACE scores were excellent risk stratification tools for mortality in the short term. The prognostic performance of each risk score was almost similar in the long term, but the CRUSADE score might be a superior risk stratification tool in the longer term than 3 years.

## Data Availability

The datasets presented in this article are not readily available because the deidentified participant data will not be shared. Requests to access the datasets should be directed to takatokuaizawa@hmedc.or.jp.
